# Supplementary figures and images for: The role of Gdf5 regulatory regions in development of hip morphology
Source: PLoS One. 2018 Nov 2;13(11):e0202785. doi: 10.1371/journal.pone.0202785 (PMC6214493; doi:10.1371/journal.pone.0202785)

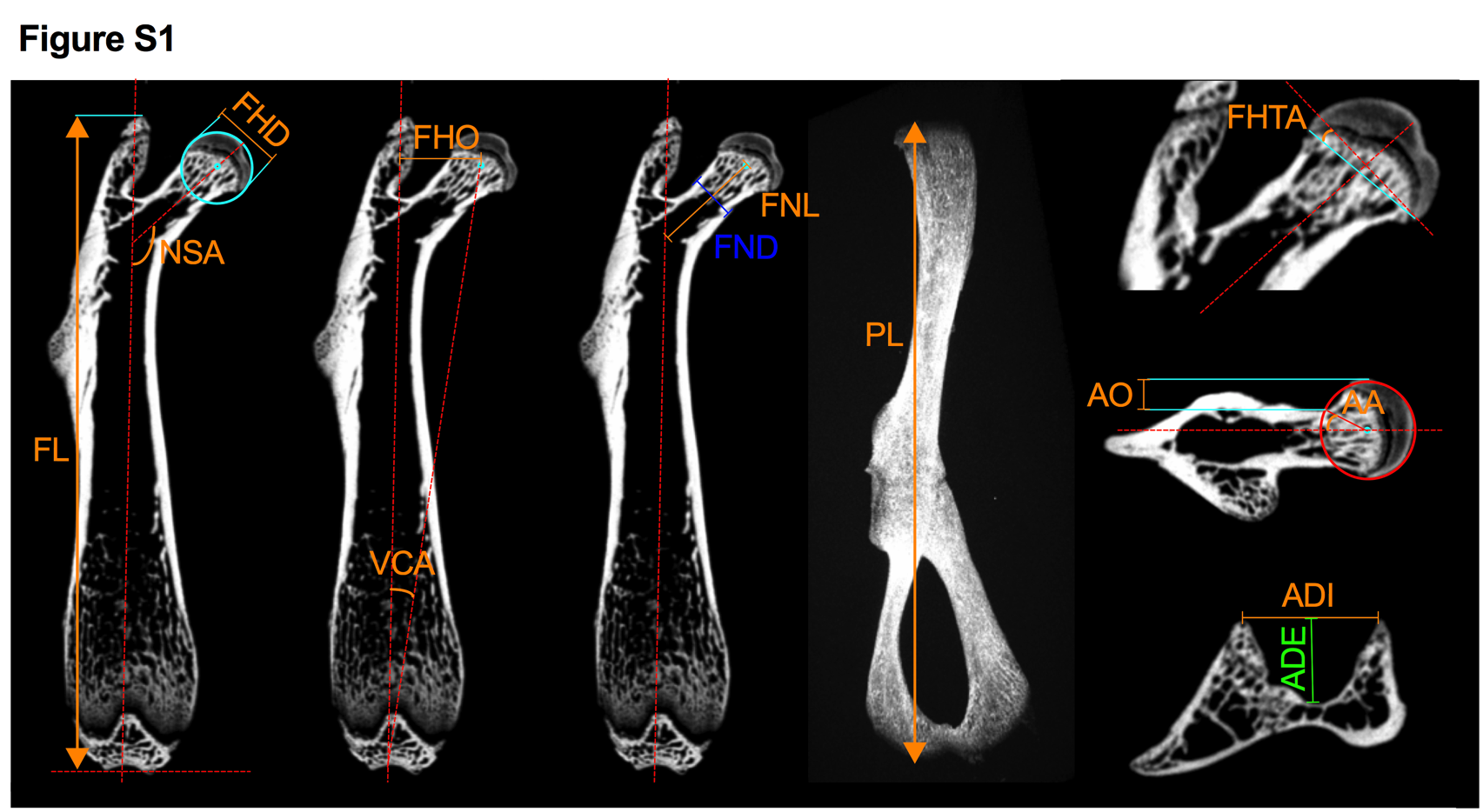

Supplement: S1 Fig — Measurements were conducted on microCT images acquired at 12 μm3 isotropic voxel size, 70 kVp peak x-ray tube intensity, 114 mA x-ray tube current, and 200 ms integration time. Femoral length (FL), femoral head diameter (FHD), femoral head offset (FHO), femoral neck length (FNL), femoral neck diameter (FND), valgus cut angle (VCA), neck shaft angle (NSA), femoral head tilt angle (FHTA), alpha angle (AA), anterior offset (AO), pelvis length (PL), acetabular diameter (ADI) and acetabular depth (ADE). (TIFF) [file pone.0202785.s001.tiff]

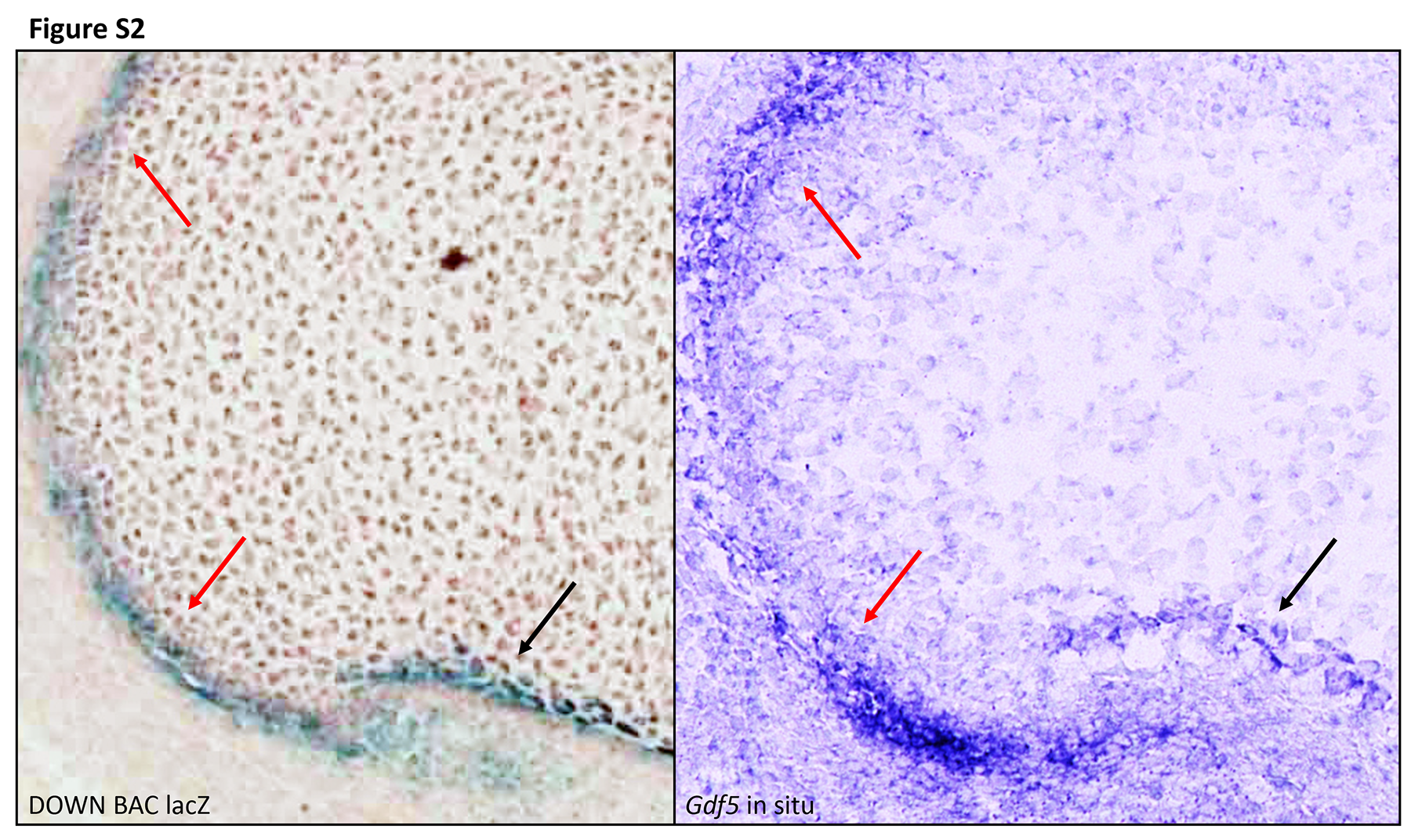

Supplement: S2 Fig — Left image shows a histological section of a portion of the mouse hip revealing expression of lacZ driven by the Downstream BAC in chondrocytes of the proximal femoral head/acetabulum (red arrows) and inferior femoral neck perichondrium (black arrows), counter-stained with nuclear fast red. The image on the right is an adjacent histological section revealing endogenous Gdf5 expression in the same domains but as assessed using in situ hybridization. (TIFF) [file pone.0202785.s002.tiff]
