# Supplementary material for: The role of Gdf5 regulatory regions in development of hip morphology
Source: PLoS One. 2018 Nov 2;13(11):e0202785. doi: 10.1371/journal.pone.0202785 (PMC6214493; doi:10.1371/journal.pone.0202785)
Supplement: S1 Table — All outcome measures were defined as continuous variables (N = 5 per genotype). Normally distributed data were compared using Analysis of Variance (ANOVA) with a post hoc Tukey correction for multiple comparisons. Non-normally distributed data were compared between the groups using Kruskal-Wallis test with a Benjamini Hochberg post hoc correction for multiple comparisons. P values are two-sided and the statistical significance was assessed at alpha = 0.05 for all the comparisons. (DOCX) [file pone.0202785.s003.docx]

**Table S1:** Differences in key morphologic features of the hip joint at 8 weeks. All outcome measures were defined as continuous variables (N=5 per genotype). Normally distributed data were compared using Analysis of Variance (ANOVA) with a post hoc Tukey correction for multiple comparisons. Non-normally distributed data were compared between the groups using Kruskal-Wallis test with a Benjamini Hochberg post hoc correction for multiple comparisons. P values are two-sided and the statistical significance was assessed at alpha = 0.05 for all the comparisons.

| **Anatomical Index** | **Genotype** | **Mean ± SD** | *** Pairwise Comparisons (P-Value)** | | | **** F or H Statistic** | ***** Test of Normality** |
| --- | --- | --- | --- | --- | --- | --- | --- |
|  |  |  | *bp/+* | *bp/bp* | *bp/bp; DOWN-BAC* |  |  |
| Femoral Length (mm) | *bp/+* | 13.57±0.38 |  |  |  | H=14.3 | P=0.038 |
|  | *bp/bp* | 10.82±0.45 | 0.014 |  |  |  |  |
|  | *bp/bp; DOWN-BAC* | 13.77±0.64 | 0.957 | 0.014 |  |  |  |
|  | *bp/bp; UP-BAC* | 10.86±0.67 | 0.014 | 0.957 | 0.014 |  |  |
| Femoral Head Diameter (mm) | *bp/+* | 1.50±0.03 |  |  |  | F=14.3 | P=0.305 |
|  | *bp/bp* | 1.30±0.11 | 0.001 |  |  |  |  |
|  | *bp/bp; DOWN-BAC* | 1.46±0.03 | 0.814 | 0.007 |  |  |  |
|  | *bp/bp; UP-BAC* | 1.27±0.07 | <0.001 | 0.948 | 0.002 |  |  |
| Femoral Head Offset (mm) | *bp/+* | 1.85±0.20 |  |  |  | F=15.5 | P=0.824 |
|  | *bp/bp* | 1.32±0.15 | <0.001 |  |  |  |  |
|  | *bp/bp; DOWN-BAC* | 1.75±0.08 | 0.662 | 0.001 |  |  |  |
|  | *bp/bp; UP-BAC* | 1.53±0.06 | 0.008 | 0.0116 | 0.074 |  |  |
| Femoral Neck Length (mm) | *bp/+* | 2.29±0.23 |  |  |  | F=7.4 | P=0.982 |
|  | *bp/bp* | 1.82±0.27 | 0.009 |  |  |  |  |
|  | *bp/bp; DOWN-BAC* | 2.23±0.13 | 0.850 | 0.023 |  |  |  |
|  | *bp/bp; UP-BAC* | 1.89±0.09 | 0.023 | 0.850 | 0.040 |  |  |
| Femoral Neck Diameter (mm) | *bp/+* | 1.02±0.04 |  |  |  | F=16.8 | P=0.455 |
|  | *bp/bp* | 0.89±0.04 | <0.001 |  |  |  |  |
|  | *bp/bp; DOWN-BAC* | 1.01±0.02 | 0.993 | 0.001 |  |  |  |
|  | *bp/bp; UP-BAC* | 0.89±0.05 | 0.001 | 0.998 | 0.001 |  |  |
| Valgus Cut Angle (degrees) | *bp/+* | 8.68±0.95 |  |  |  | H=6.0 | P=0.045 |
|  | *bp/bp* | 7.60±0.66 | 0.087 |  |  |  |  |
|  | *bp/bp; DOWN-BAC* | 8.18±0.61 | 0.325 | 0.325 |  |  |  |
|  | *bp/bp; UP-BAC* | 8.28±0.46 | 0.325 | 0.325 | 0.851 |  |  |
| Neck Shaft Angle (degrees) | *bp/+* | 127.4±4.4 |  |  |  | F=1.8 | P=0.160 |
|  | *bp/bp* | 132.4±4.1 | 0.220 |  |  |  |  |
|  | *bp/bp; DOWN-BAC* | 127.9±4.2 | 0.996 | 0.306 |  |  |  |
|  | *bp/bp; UP-BAC* | 128.0±2.6 | 0.995 | 0.313 | 0.999 |  |  |
| Femoral Head Tilt Angle (degrees) | *bp/+* | 1.9±1.3 |  |  |  | H=3.9 | P=0.007 |
|  | *bp/bp* | 3.7±2.0 | 0.465 |  |  |  |  |
|  | *bp/bp; DOWN-BAC* | 2.5±0.9 | 0.694 | 0.616 |  |  |  |
|  | *bp/bp; UP-BAC* | 5.9±4.4 | 0.430 | 0.694 | 0.465 |  |  |
| Alpha Angle (degrees) | *bp/+* | 23.42±1.08 |  |  |  | F=9.6 | P=0.731 |
|  | *bp/bp* | 28.9±2.9 | 0.038 |  |  |  |  |
|  | *bp/bp; DOWN-BAC* | 23.5±4.5 | 0.999 | 0.040 |  |  |  |
|  | *bp/bp; UP-BAC* | 31.5±2.1 | 0.002 | 0.537 | 0.003 |  |  |
| Anterior Offset (mm) | *bp/+* | 0.42±0.04 |  |  |  | F=10.4 | P=0.427 |
|  | *bp/bp* | 0.34±0.03 | 0.037 |  |  |  |  |
|  | *bp/bp; DOWN-BAC* | 0.44±0.05 | 0.898 | 0.009 |  |  |  |
|  | *bp/bp; UP-BAC* | 0.32±0.03 | 0.005 | 0.769 | 0.001 |  |  |
| Anteversion (degrees) | *bp/+* | 20.3±4.9 |  |  |  | F=1.6 | P=0.077 |
|  | *bp/bp* | 24.9±6.8 | 0.506 |  |  |  |  |
|  | *bp/bp; DOWN-BAC* | 25.6±5.3 | 0.393 | 0.997 |  |  |  |
|  | *bp/bp; UP-BAC* | 20.1±3.4 | 0.999 | 0.472 | 0.363 |  |  |
| Pelvis Length (mm) | *bp/+* | 16.08±0.57 |  |  |  | H=11.2 | P=0.043 |
|  | *bp/bp* | 15.89±0.29 | 0.789 |  |  |  |  |
|  | *bp/bp; DOWN-BAC* | 16.22±0.48 | 0.789 | 0.731 |  |  |  |
|  | *bp/bp; UP-BAC* | 13.89±0.59 | 0.019 | 0.043 | 0.017 |  |  |
| Acetabular Diameter (mm) | *bp/+* | 1.72±0.08 |  |  |  | F=14.2 | P=0.261 |
|  | *bp/bp* | 1.45±0.07 | <0.001 |  |  |  |  |
|  | *bp/bp; DOWN-BAC* | 1.70±0.04 | 0.972 | 0.001 |  |  |  |
|  | *bp/bp; UP-BAC* | 1.54±0.10 | 0.009 | 0.314 | 0.021 |  |  |
| Acetabular Depth (mm) | *bp/+* | 0.92±0.01 |  |  |  | F=9.9 | P=0.325 |
|  | *bp/bp* | 0.73±0.11 | 0.004 |  |  |  |  |
|  | *bp/bp; DOWN-BAC* | 0.89±0.06 | 0.884 | 0.017 |  |  |  |
|  | *bp/bp; UP-BAC* | 0.73±0.07 | 0.003 | 0.999 | 0.014 |  |  |

* Adjusted P value. ** F-Statistic for ANOVA and H-Statistic for non-parametric Kruskal-Wallis test. *** Shapiro-Wilk test, P > 0.05 indicates normal distribution.
