# Supplementary material for: The role of Gdf5 regulatory regions in development of hip morphology
Source: PLoS One. 2018 Nov 2;13(11):e0202785. doi: 10.1371/journal.pone.0202785 (PMC6214493; doi:10.1371/journal.pone.0202785)
Supplement: S2 Table — (DOCX) [file pone.0202785.s004.docx]

**Table S2:** BAC prenatal and postnatal expression patterns.

| **Anatomical Site** | **E17** | | **6 Months** | |
| --- | --- | --- | --- | --- |
|  | ***UP-BAC*** | ***DOWN-BAC*** | ***UP-BAC*** | ***DOWN-BAC*** |
| **Pelvic Girdle, Hip-Joint** |  |  |  |  |
| Ilium Growth Plate | NO | YES | NO | YES-WEAK* |
| Pubis Growth Plate | NO | YES | NO | YES-WEAK* |
| Ischium Growth Plate | NO | YES | NO | YES-WEAK* |
| Acetabulum: | YES-WEAK | YES | NO | YES |
| Labrum | YES-WEAK | YES | NO | YES-RIM ONLY |
| Lig. Teres orig. | YES | YES | NO | NO |
| Lunate Surface | YES-WEAK | YES | NO | YES |
| Lig. Transverse | NO | YES | NO | NO |
| Lig. Iliofemoral orig. | NO | YES | NO | NO |
| Lig. Pubofemoral orig. | NO | YES | NO | NO |
| Lig. Ischiofemoral orig. | NO | YES | NO | NO |
| **Proximal Femur** |  |  |  |  |
| Femoral Head Region: | YES-WEAK | YES | NO | YES |
| Articular Cartilage | YES-WEAK | YES | NO | YES |
| Growth Zone | NO | YES | NO | NO |
| Lig. Teres insert. | YES-WEAK | YES | NO | NO |
| Lig. Iliofemoral insert. | NO | YES | NO | NO |
| Lig. Pubofemoral insert. | NO | YES | NO | NO |
| Lig. Ischiofemoral insert. | NO | YES | NO | NO |
| Femoral Neck | NO | YES | NO | NO |
| Trochanteric Region | NO | YES | NO | NO |

* Background staining can be observed at times in negative control mice.
